# Alpha-Synuclein Neurobiology in Parkinson’s Disease: A Comprehensive Review of Its Role, Mechanisms, and Therapeutic Perspectives

**DOI:** 10.3390/brainsci15121260

**Published:** 2025-11-25

**Authors:** Jamir Pitton Rissardo, Andrew McGarry, Yiwen Shi, Ana Leticia Fornari Caprara, George T. Kannarkat

**Affiliations:** 1 Neurology Department, Cooper University Hospital, Camden, NJ 08103, USA; 2 Cooper Medical School, Rowan University, Camden, NJ 08103, USA; 3 Department of Neurology, University of Pennsylvania, Philadelphia, PA 19104, USA; 4 Parkinson’s Disease Research, Education and Clinical Center, Corporal Michael J. Crescenz Department of Veterans Affairs Medical Center, Philadelphia, PA 19104, USA

**Keywords:** synucleinopathy, co-pathology, neurodegeneration, immunotherapy, biomarkers, prion-like spread

## Abstract

Parkinson’s disease (PD) is a progressive neurodegenerative disorder characterized by the loss of dopaminergic neurons in the substantia nigra (SN) and the presence of intracellular α-synuclein (αSyn) aggregates known as Lewy bodies (LB). αSyn, a presynaptic protein, is believed to play a crucial role in synaptic function, neurotransmitter release, and neuronal plasticity. However, its misfolding and aggregation are thought to be central to PD pathogenesis. This review provides a comprehensive analysis of αSyn’s role in PD, exploring its normal physiological functions, pathological mechanisms, and therapeutic potential. The pathological transformation of αSyn involves structural alterations that promote oligomerization and fibrillization, leading to toxic gain-of-function effects. These aggregates disrupt cellular homeostasis through mechanisms including mitochondrial dysfunction, oxidative stress, lysosomal impairment, and endoplasmic reticulum stress. Furthermore, pathogenic αSyn is thought to exacerbate neurodegeneration via prion-like spread along interconnected neuronal circuits. Emerging evidence highlights the frequent co-occurrence of other proteinopathies, such as tau and amyloid-β, which may synergistically accelerate disease progression. Targeting αSyn has emerged as a potential therapeutic strategy. Approaches such as immunotherapy, small-molecule inhibitors, gene silencing, and modulation of protein degradation pathways (e.g., autophagy and proteasomal systems) are actively being explored. Additionally, lifestyle-based interventions, particularly exercise, have shown neuroprotective effects, potentially mediated by irisin—a myokine implicated in protein clearance and synaptic resilience—underscoring the importance of multimodal strategies in PD management.

## 1. Introduction

An early description of Parkinson’s disease (PD) appeared in ancient Greek and Roman literature 3rd millennium BC [1]. In 1817, the first clinical symptoms of the disease then called shaking palsy were broadly described by James Parkinson. Jean-Martin Charcot named the disease after Parkinson in 1867 [2]. Almost a century later, in 1912, abnormal protein aggregates, called Lewy bodies (LB), were identified in neurons of people with Parkinson’s disease (PWP). In 1917, Konstantin Nikolaevitch Tretiakoff linked the cardinal motor symptoms of PD to the loss of neurons in a small brain region called the substantia nigra (SN) [3]. The year 1997 marked a major milestone in the knowledge and understanding of PD with the discovery that LB are in part made of a protein called αSyn [4].

While αSyn aggregation is the hallmark of PD pathology thought to be a major element of pathogenesis, there remain many knowledge gaps in understanding its exact pathogenic role. αSyn is expressed abundantly in neurons among other cell types throughout the body yet its precise functions remain uncertain. It is a dynamic protein adopting different conformations that likely correspond to different functions of the protein. These forms may interact, combine, and form αSyn multimers, which are transient (form and come undone continuously) [5]. αSyn-related neuronal metabolism is characterized by efficient degradation and recycling systems such as molecular chaperones and the proteasome, which balance αSyn forms. In certain contexts, this equilibrium may be disrupted leading to abundant formation of αSyn multimers, ultimately leading to the irreversible formation of fibrils occupying spaces in neurons and leading to the development of LB.

The accumulation of toxic multimers and fibrils is thought to lead to abnormal synaptic transmission and eventual cell death, i.e., the loss of dopaminergic neurons in the SN leading to the emergence of PD motor symptoms [6]. However, PD is not limited to motor dysfunction. Non-motor symptoms—such as autonomic disturbances, cognitive impairment, and mood disorders—are likely attributable to neurodegeneration in other brain regions beyond the SN [7]. Moreover, clinical heterogeneity in both motor and non-motor symptom onset and progression is a defining feature of αSyn-associated diseases, reflecting the complex and multifocal nature of αSyn pathology.

## 2. αSyn in PD

In 1912, German-born American neurologist Fritz Heinrich Lewy, also known as Friedrich Heinrich Lewy, was the first to find intraneuronal eosinophilic proteinaceous inclusions in the brain, which were later termed LB [8]. They are intracellular inclusions primarily characterized by a laminar core surrounded by a peripheral halo, both containing aggregated αSyn fibrils. In addition to αSyn, LBs also contain other components such as ubiquitin, neurofilament proteins, and lipids, reflecting their complex composition and potential involvement in cellular degradation pathways. In 1997, αSyn has been demonstrated to be a major component of LB and Lewy neurites [9]. And, subsequently these LB have been described in various brain regions affected in PD, including the locus coeruleus and SN [10]. More recently, high-resolution microscopy and lipodomic studies have highlighted that LB pathology in PD can include crowded organelles and lipid membranes [11].

Beyond the CNS, αSyn pathology has been consistently reported in peripheral organs, including the autonomic nervous system, gastrointestinal tract, salivary glands, and skin [12]. These findings have fueled interest in peripheral tissue biopsies as potential diagnostic tools for PD. For example, αSyn aggregates have been detected in SMG and colon biopsies, and more recently, skin biopsies have emerged as a promising, minimally invasive method for detecting phosphorylated αSyn in peripheral nerves. The Systemic Synuclein Sampling Study (S4) demonstrated the feasibility and relative safety of obtaining multiple peripheral tissues—skin, colon, and submandibular gland—alongside biofluids for αSyn analysis in PD patients [13]. While these approaches hold promise for early diagnosis and biomarker development, significant gaps remain: we do not fully understand why peripheral αSyn pathology occurs, nor the temporal relationship between its appearance in peripheral tissues and the brain. Whether peripheral αSyn deposition represents an early initiating event, a parallel process, or a downstream consequence of central pathology remains unresolved.

Another study in 1997 discovered the first inherited PD-causing mutation of αSyn, Ala53Thr (A53T) mutation, in the long arm of chromosome 4 of a kindred from Contursi (Southern Italy) with multiple affected members with autosomal dominant pattern of inheritance, and some of the family members had atypical clinical features [14]. In 2003, Singleton and colleagues describe the association of multiplication of αSyn with PD. The triplication of the gene is associated with early-age of onset and more severe phenotype suggesting an association between αSyn levels and disease risk [15]. Multiple missense mutations have been described in PD: A30P, A53E, A53T, E46K, G51D, and H50Q (Table 1) [16,17,18,19,20,21,22,23].

Multiple genome-wide association study (GWAS) in different ethnic populations have identified αSyn to be the top hit association with sporadic PD [24,25]. Maraganore et al. led the International Consortium of Genetic Epidemiology of PD (GEO-PD) to validate the association of αSyn promoter region and the SNP (single nucleotide polymorphism) dinucleotide repeat sequence (REP1), in which particularly longer alleles with more than 263 base-pair were associated with increased risk of sporadic PD [26]. Subsequently methylation that can regulate αSyn expression was shown to be decreased in PWP [27]. Various cross-sectional and longitudinal studies have shown that a carrier of SNP REP1 has high risk of motor and non-motor progression of PD, particularly cognitive impairment [28] and executive function [29]. In addition, αSyn mRNA expression levels have been shown to be upregulated or higher in people with cognitive problems [30]. Moreover, mutations in the distal enhancer (rs356168) [31] and age at onset (rs356203) [32] were associated with poor cognition and increased αSyn levels. Interestingly, the total αSyn protein levels in the CSF of patients with PD is decreased, but the relative levels of oligomeric and phosphorylated forms and their correlation with disease progression remain uncertain [33]. In this context, oligomeric and phosphorylated αSyn are already seen in other conditions such as MSA and AD [34].

### 2.1. The αSyn Protein

**Figure 1:**
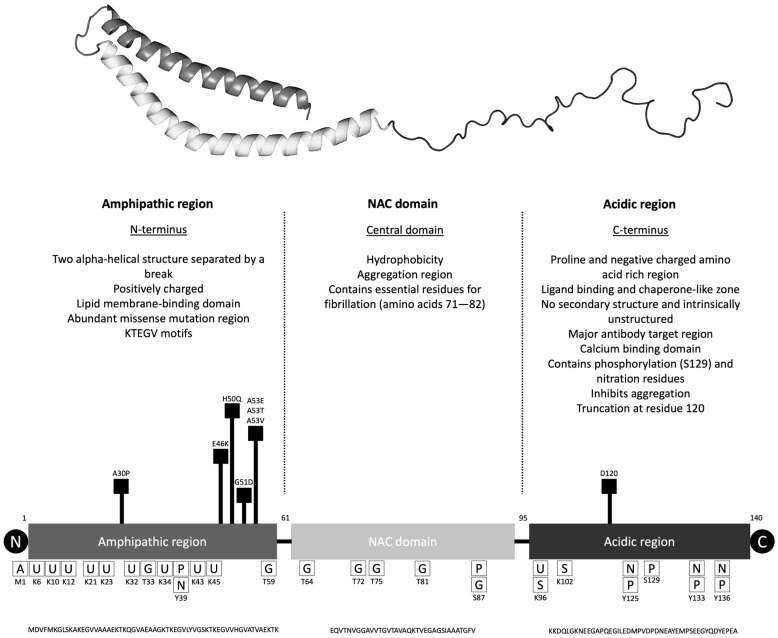
Structure of αSyn. In the figure are represented the domains, amino acid sequence, missense mutations, and major post-translational modifications sites of αSyn. Abbreviations: A, acetylation; N, nitration; P, phosphorylation; S, SUMOylation; U, ubiquitination; G, O-GlcNAcylation.

αSyn was first identified in electric fish (Torpedo californica) and derives its name from localization in presynaptic nerve terminals [35]. It is a 140-amino acid protein with three main regions called N-terminal amphipathic region, central non-Aβ component (NAC) region, and C-terminal acidic region (Figure 1 and Table 2). The N-terminal has the affinity to bind to lipid membranes, and most of the missense mutations are localized in this region. The NAC region is thought to play a role in aggregation due to its hydrophobicity and the intrinsically unstructured C-terminal region is important for membrane binding but also aggregation. Of note, calcium concentration changes can affect how αSyn binds to lipid membranes [36]. The association of αSyn with lipid membranes is hypothesized to regulate its ability to form multimers and aggregate into fibrils.

αSyn is highly expressed in the brain, especially in the presynaptic terminal. Also, it is found in the spinal cord, peripheral nerves, and the enteric nervous system. There are lower levels of αSyn expression in organs such as the heart, muscle, blood cells, liver, kidneys, and lungs. Interestingly, red blood cells are the major source (99%) of αSyn in blood, only 0.1% is contained in the plasma [57]. In the synaptic terminal, αSyn is found in the presynaptic terminal (synaptic vesicle, active zones), cytoplasm, membrane-bound forms (plasma membrane, intracellular membranes), and nucleus [58].

αSyn can undergo multiple posttranslational modifications, in particularly acetylation, nitration, phosphorylation, ubiquitination, and truncation (mainly in the C-terminal) as well as non-enzymatic glycation [59]. More importantly any modification of the protein, particularly in the NAC region and C-terminal region can affect its aggregation affinity [60]. From a clinical standpoint, phosphorylation of αSyn at serine-129 (pS129) is a widely recognized pathological hallmark of PD, with approximately 90% of αSyn found in LB being phosphorylated at this site [61,62]. Notably, pS129 has been detected in various body fluids and brain tissue, suggesting its potential utility as a biomarker. Recent studies also highlight its physiological role in regulating synaptic activity and neuronal signaling [63]. Additionally, phosphorylation at tyrosine-39 (p-Tyr39) has emerged as another important post-translational modification, implicated in modulating αSyn aggregation and toxicity, further underscoring the complexity of αSyn pathology in PD [64].

Under physiological conditions, αSyn is predominantly an intrinsically disordered monomer [65]. In this context, some studies have shown that αSyn may also exist as a helically folded tetramer or multimer in healthy cells [66], which appears to be more stable and resistant to aggregation, suggesting a potential protective role in healthy neurons [67]. However, αSyn can adopt various conformations and transition into oligomeric species, which are believed to be more pathogenic. These oligomers can further aggregate into insoluble fibrils, a hallmark of synucleinopathies such as PD [68]. The fibril with αSyn monomers and α-B-crystallin can adhere to a neurofilament, and undergo ubiquitination to form the LB [69].

αSyn can be unfolded, particularly the oligomeric species by chaperone proteins, entering the off pathway without aggregation and resulting in important physiology and pathophysiology properties with therapeutic implications. αSyn monomers are stabilized by their propensity to attach to curved membranes of vesicles; however, pathogenic multimers may induce membrane damage and pull together multiple proteins and enzymes to form the pale bodies that are the precursors of LB [70].

Recent evidence suggests that these physiological tetramers are not confined to neurons but are also present in peripheral tissues, including blood cells, where αSyn is highly abundant in erythrocytes. Importantly, studies have demonstrated that the ratio of aggregation-resistant tetramers to monomers is significantly reduced in both familial and sporadic PD patients compared to healthy controls, and even in asymptomatic carriers of SNCA mutations such as G51D [71]. This reduction in tetramer levels may precede clinical symptom onset, supporting the hypothesis that destabilization of αSyn multimers is an early pathogenic event. Consequently, measuring tetramer/monomer ratios in blood has been proposed as a promising, minimally invasive biomarker for early detection and disease monitoring in PD [71].

The activity and pathogenic potential of αSyn are influenced by two key factors: its morphology and its aggregation kinetics. Super-resolution microscopy and small-angle X-ray scattering (SAXS) studies have identified several distinct oligomeric forms of αSyn. Notably, many of these oligomers exhibit an annular (ring-like) shape with a central depression, which may be relevant to their membrane-disruptive properties [72]. As aggregation progresses, αSyn can form protofilaments—linear assemblies with relatively uniform height. These protofilaments can intertwine to form protofibrils, which in turn can further associate into mature fibrils. Importantly, the final morphology of αSyn fibrils is heavily influenced by the structural characteristics of the protofilaments from which they are formed. This hierarchical assembly process contributes to the structural diversity observed in αSyn fibrils across different synucleinopathies.

### 2.2. Prion-like Spread of αSyn in Cell Cultures and Animal Models

In multiple models, αSyn shows the ability to spread in a prion-like fashion, templating the misfolding and aggregation of monomeric αSyn, and induce LB-like pathology. In vitro, the overexpression of wild type or mutant aggregation-prone αSyn can lead to development of inclusions. The cellular models already studied include yeast, non-neuronal (HEK293 and H4), primary neurons, differentiated immortalized cells (PC12, SH-SY5Y, and LUHMES-Lund human mesencephalic), and patient derived (fibroblasts/peripheral blood mononuclear cells, iPSCs-induced pluripotent stem cells, and iNeurons) [73]. The formation of αSyn multimers and fibrils in these models can be associated with various pathological features including mitochondrial dysfunction, proteasome impairment, and oxidative stress leading to cell death. pS129 and resistance solubilization by detergents can be used as a readout for αSyn pathology [74]. Depending on the model, this pathology can be induced by extracellular addition of αSyn fibrils but sometimes a lipophilic carrier molecule must be used to allow cellular entry [75].

In patient-derived αSyn mutant midbrain organoids in 3D cell culture, LB-like inclusions visualized by EM or staining for hematoxylin/eosin, αSyn, and ubiquitin four to six months after culture. The number of LB is typically low and very similar to post-mortem human studies [76]. Interestingly, the overexpression of αSyn in the Drosophila αSyn model can produce a phenotype of PD (loss of dopamine and dopamine cluster, increased mortality, and LB inclusions), and when a drug preventing αSyn aggregation was applied, there was a significant difference on day 40 regarding movements of the limbs [77].

There are three main αSyn mouse models: αSyn transgenic (Tg) mouse model, αSyn adeno-associated virus (AAV) injection model mouse, and the αSyn preformed fibril injection model (Table 3) [78]. The αSyn Tg model involves genetic overexpression of αSyn, facilitating long-term assessments of phenotype-pathology correlations. However, while many Tg mouse models—including both wild-type and mutant αSyn lines—display varying phenotypes with respect to inclusion type, distribution, and associated neurodegeneration, they generally do not fully recapitulate the hallmark LB pathology observed in human disease. Moreover, the extent of αSyn propagation differs across models and remains limited compared to human pathology.

Neuronal involvement is variable, and while motor symptoms are inconsistent, immune dysregulation is present [79]. In Tg mouse αSyn models, the expression of αSyn either the wild type or the mutant is associated with varied motor disability with age of onset ranging from two months to 12 months (Table S1) [80,81,82,83,84,85,86]. These mice also demonstrate αSyn inclusions. The A53T Tg mouse has a progressive neuropathology and is the line that most frequently reported in the literature [87]. In contrast, the αSyn AAV mouse, designed for short-term, high-throughput evaluations, shows robust αSyn expression and neuronal involvement but does not develop LB pathology or exhibit αSyn spreading [88]. This model presents pronounced motor symptoms with a stronger immune response compared to the Tg model in a shorter time frame, making it suitable for therapeutic screening studies [89].

The αSyn preformed fibril mouse, generated via inoculation with LB extracts, provides a more physiologically relevant model of synucleinopathy by allowing endogenous αSyn to misfold and aggregate. Unlike other models, it develops LB pathology, exhibits extensive αSyn spreading, and manifests both motor and non-motor symptoms, making it valuable for studying disease progression. Neuronal involvement is evident, and the immune response is comparable to that of the AAV model. The heterogeneity of αSyn species within LB extracts further enhances the translational relevance of this model for investigating αSyn pathology and therapeutic interventions. Given these characteristics, the αSyn preformed fibril model is the most widely used in preclinical research on PD and related synucleinopathies [90].

### 2.3. Molecular Pathways Disrupted by αSyn Mutations

The accumulation of αSyn may disrupt the molecular mechanisms mirroring the underlying pathological hallmarks in PD. Some of molecular pathway’s abnormalities caused by αSyn protein aggregation are mitochondrial dysfunction, autophagy impairment, exome propagation, astrocyte activation, axon transport inhibition, synapse dysfunction, microglial cell activation, lysosome dysfunction, and proteasome inhibitions.

#### 2.3.1. Synaptic Dysfunction

αSyn plays a crucial role in maintaining synaptic integrity and regulating vesicle trafficking. This has been demonstrated in both overexpression and knockout models, where disruption of αSyn levels leads to impaired synaptic function [91]. Under physiological conditions, monomeric αSyn is particularly important for synaptic vesicle cycling, including the mobilization and maintenance of the vesicle reserve pool. Its interaction with synaptic vesicle membranes helps regulate neurotransmitter release and supports efficient synaptic transmission. It is also integral to regulation of the SNARE (soluble N-ethylmaleimide-sensitive factor attachment protein receptors) complex that opens the synaptic pore leading to more effective attachment and release of the neurotransmitter [92]. Therefore, aggregation can affect neurotransmitter release and synaptic function by abnormalities (pounding and physically blocking) in vAMP2 (vesicle-associated membrane protein 2) and vMAT2 (vesicle monoamine transporter type 2) function [93]. Aggregates of αSyn can therefore disrupt the structure and function of synapses, synaptic loss, and neuronal connectivity [94].

#### 2.3.2. Mitochondrial Dysfunction

αSyn aggregates are also present in mitochondria, which may impair their function and contribute to neuronal cell death. Increasing aggregate concentrations can lead to production of reactive oxygen species exacerbating oxidative damage to cellular components such as lipids, proteins, and DNA. During physiological conditions, various cytosolic proteins enter the mitochondria by the VDAC (voltage-dependent anion channel) and TOM (translocase of the outer membrane) complexes [95]. But, αSyn, if phosphorylated or forming oligomers, can potentially block these channels, in particular the TOM 20 complex, through spectrin interaction with actin component of the cytoskeleton [96]. Blockage of the cytosolic protein entrance leads to an increase in oxidative oxygen species that can enhance αSyn aggregation entering in a vicious cycle and impairing complex 1, DNA polymer of the mitochondria, and ATP (adenosine triphosphate) synthetase function. In addition, it can also affect the function of cardiolipin translocation when reactive oxygen species levels are too high in the outer membrane [97]. These events increase the mitochondrial potential, leading to PTP (permeability transition pore) opening and exudation of cytochrome c and influx of solute and water, causing dyshomeostasis and ballooning out of mitochondria culminating in cell death [98].

#### 2.3.3. Neuroinflammation

Aggregated αSyn activates microglia, initiating a chronic inflammatory response that contributes to neuronal damage. Activated microglia release pro-inflammatory cytokines such as IL-1β, TNF-α, and IL-6, which exacerbate neuronal injury and perpetuate a toxic environment. A key mediator of this response is the NLRP3 inflammasome, a multiprotein complex that senses cellular stress and promotes the maturation of IL-1β and IL-18. Inhibition of microglial activation and NLRP3 inflammasome signaling has been proposed as a therapeutic strategy to reduce αSyn pathology and neuroinflammation [99].

Microglia also form F-actin-dependent intracellular networks that facilitate the engulfment and degradation of αSyn aggregates. These networks can transfer αSyn fibrils to mitochondria for degradation, a process that is impaired by PD-associated mutations [100]. Interestingly, activated microglia may also exchange healthy mitochondria with neighboring cells to mitigate oxidative stress and maintain cellular homeostasis [101].

In mouse models, lentiviral overexpression of αSyn specifically in microglia has been shown to induce dopaminergic neurodegeneration, even in the absence of detectable αSyn accumulation, highlighting the inflammatory potential of microglial αSyn expression [102].

#### 2.3.4. Lipid Accumulation in Dopamine Neurons

αSyn interacts with membrane phospholipids and free fatty acids. The N-terminal altered lipid-protein complexes affect binding to synapse and mitochondrial membrane and oligomerization. One paradigm could be that αSyn monomers adopt different conformation such as a broken helix or double helix, which pull multimers together in a physiological situation. However, in a pathogenic condition, αSyn can adopt or partially adopt conformations that exposes the NAC region and promote lipid induced aggregate formation [103]. The situation depends on the lipid composition, the lipid and αSyn differences, and the fraction of αSyn in the membranous cytosolic form which may determine whether αSyn enters a pathogenic or physiological pathway [104].

#### 2.3.5. Disruption of Cellular Homeostasis

αSyn aggregates disrupt calcium homeostasis in neurons, leading to excitotoxicity and neuronal death. This was confirmed in midbrain dopaminergic neurons by post-mortem studies in individuals with familial PD [105]. It was recently proposed that there is likely a dysfunction in the MAM system characterized by the interaction between the mitochondria and the endoplasmic reticulum membrane leading to accumulation of calcium [106]. Lysosomal dysfunction can be impaired by accumulation of αSyn, contributing to the increase in concentration of toxic substance within neurons [107].

There is also impairment of endoplasmic reticulum-Golgi transport. Misfolded αSyn can overwhelm the endoplasmic reticulum capacity to process proteins properly, leading to endoplasmic reticulum stress. Chronic endoplasmic reticulum stress from persistent αSyn aggregation sustained UPR (unfolded protein reaction) activation and neurodegeneration [108]. Finally, αSyn aggregates can physically disrupt endoplasmic reticulum function and Rab1, affecting calcium homeostasis and protein trafficking [109].

#### 2.3.6. Impaired Autophagy and Proteasome

αSyn is degraded by both autophagy and the proteasome mechanisms [110]. αSyn aggregates inhibit autophagy, leading to accumulation of toxic proteins and damaged organelles, which can be observed by the impairment of SNAP29 (synaptosome-associated protein 29) function [111]. αSyn may impair the ubiquitin-proteasome system (UPS) reducing the cell’s ability to degrade misfolded proteins, cellular stress, and apoptosis. Interestingly, αSyn through autophagy and proteasome mechanisms may regulate the viability during the stationary phase in which the rate of cell division equals the rate of cell death [112] to promote neurodegeneration in mouse models of PD [113].

αSyn aggregates can accumulate in autophagy deficient dopaminergic neurons in aged mice; notably, the accumulation was only observed in 20 months of the study and young neurons were not affected by the aggregates [114]. Another study observed that neuron-release of αSyn induces microglial morphology changes and is engulfed by microglia in vivo. Interestingly, no overt neurodegeneration or behavioral deficits were observed in Thy1-human αSyn transgenic mice; instead, the human αSyn appeared to interact with and internalize pathological αSyn species, suggesting a potential role in the propagation of misfolded protein aggregates. Also, there were significant abnormalities observed in the microglia with AAV-9-αSyn but not with AAV9-GFP (green fluorescent protein). Extracellular αSyn activates TLR (toll-like receptor) signaling and autophagy receptor SQSTM1/p62 expression in microglia. Internalized αSyn co-localizes with autophagosome markers and disappears over time in microglia by autophagy pathways (ATG7-autophagy related 7 and ATG14-autophagy related 14) [115].

**Figure 2:**
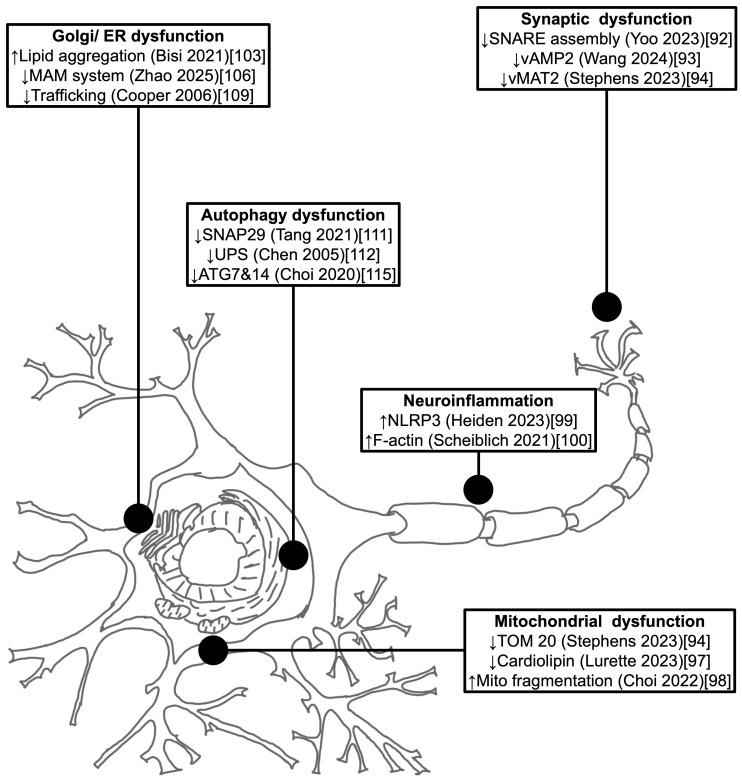
Schematic representation of a neuron highlighting major cellular dysfunctions implicated by pathogenic αSyn. Mutant αSyn promotes misfolding and aggregation into oligomers and fibrils, triggering prion-like propagation across neurons. These aggregates impair synaptic vesicle trafficking and mitochondrial function, disrupt endoplasmic reticulum homeostasis, and interfere with axonal transport. Additionally, αSyn mutations compromise protein clearance mechanisms, including chaperone-mediated autophagy and the ubiquitin–proteasome system, while activating neuroinflammatory cascades through microglial and astrocytic signaling. Collectively, these alterations converge to induce oxidative stress, calcium dysregulation, and neuronal death, highlighting the multifactorial nature of αSyn-driven neurodegeneration [92,93,94,97,98,99,100,103,106,109,111,112,115].

Microglia can react to both monomeric and oligomeric αSyn through TLR2 [116] and TLR4 [117], in the production of inflammation and response to monomeric and oligomeric forms of αSyn. Human αSyn preferentially signaling through TLR4 over TLR2 in a study with transfected HEK293T cells treated with recombinant monomeric human αSyn [115]. Consider reading Figure 2, for further understanding of the cellular dysfunctions related to pathogenic αSyn [92,93,94,97,98,99,100,103,106,109,111,112,115].

## 3. Transmission of Misfolded αSyn Protein in LB Diseases

There are shared pathogenic mechanisms among age-related neurodegenerative diseases with Aβ plaques, tau tangles, αSyn (LB), and TDP-43 inclusions [118]. Per the Braak hypothesis, LB first develop in the brainstem, then the SN, and from there appear in the entire brain. The major neurodegenerative conditions associated with αSyn pathology are PD, PDD, DLB, MSA, and AD. AD, the most common neurodegenerative disease, is typically classified as a tauopathy, but has increasingly been recognized to have αSyn co-pathology [119]. There are two main hypotheses for the spreading of αSyn. The transmission hypothesis is based on the cell-to-cell spread of LB pathology, and the strain hypothesis in the existing pathological αSyn strain can potentially explain the clinical diversity.

Initial experiments were performed with inoculation of synthetic mouse αSyn PFFs into the dorsal striatum of non-Tg mice [120]. Some of the techniques for αSyn were obtained from the prion field since it was observed that prions produced specific proteins, so it was first believed that the human protein would not function well in the mouse model. To overcome this possible species barrier the αSyn was obtained from mice. Intra-striatal injection of mouse αSyn PFFs in wildtype mice recruit endogenous αSyn to form LB and Lewy neuritis. The pathology in the SN pars compacta by αSyn aggregates can lead to impaired balance and motor coordination [121]. Therefore, αSyn PFFs initiate conversion of endogenous αSyn and their accumulation into LB and Lewy neurite-like inclusions in animal models. αSyn inclusions drive the selective loss of SN pars compact dopamine neurons, resulting in behavioral impairment reminiscent of human PD.

αSyn may form different structural conformations or strains, leading to its, different cell-type specificity, different brain region selectivity, and different rates of disease progression. Testing transmission (spread) of strains αSyn in mouse models involve extracting different αSyn strains from human brains with LB (Figure S1). Human αSyn strains are injected directly into non-Tg mouse brains to observe for spread of αSyn aggregates in the mouse brain. The authors performed ELISA, BCA, and western blood analysis of pathological (Sarkosyl-insoluble) αSyn in the patients with AD and PDD. The induction of phosphorylated αSyn pathology in mouse primary hippocampal neurons treated with LB extracts from human-brain derived αSyn is much more potent than PFFs, and this was observed in a dose-dependent manner (but not with PFFs). The quantification of phosphorylated αSyn pathology in mouse hippocampal neurons treated with human seeds showed a significant quantification, instead PFFs have a lot of pathology but did not show LB [122].

In vitro amplification of AD and PD LB αSyn, seeds were obtained from these individuals (5%) and added with αSyn monomers (95%), agitated and after four to 21 days, there was amplification of LB in a dose-dependent manner. αSyn aggregates at different times after the amplification were investigated using centrifugation-based sedimentation assay and showed similar results with no-P (without any pass), P1 (first pass), and P2 (two passes) procedures. The co-localization of phosphorylated αSyn with ubiquitin or P62 in primary hippocampal neurons treated with amplified LB from AD brains were the same. Similar findings were also observed with PK (proteinase K) resistance of LB seeds and amplified LB derived from the brains of patients with AD or patients with PDD [123].

Pathological αSyn derived from patient LB induces distinct pathology compared to PFFs in cultured neuron model. Additionally, LB αSyn is more potent than PFFs in seeding αSyn aggregation in hippocampal neurons [124]. LB aggregates can be amplified in vitro using recombinant αSyn, generating aggregates that preserve the unique pathological characteristics of the original disease-derived material. In wild-type mice, amplified LB aggregates induced αSyn pathology across multiple brain regions, with a pattern of spread distinct from that induced by PFFs [125]. These findings suggest that LB-derived αSyn aggregates elicit a unique cellular response not seen with fully recombinant fibrils. The ability to generate large quantities of amplified αSyn will advance research into PD and DLB, facilitating the development of targeted therapeutics.

### 3.1. Propagation of αSyn Pathology: In Vivo Evidence

The first evidence of αSyn propagation in humans is the demonstration of LB in grafted dopamine neurons ten to fifteen years post-fetal dopaminergic neuron transplant [126]. Those who received the transplant 24 years prior develop a greater percentage (up to 12%) of LB compared to those that received it later (12 years prior; up to 5%) [127].

Several experimental models have demonstrated the prion-like propagation of αSyn pathology. In one study, neurons were cultured in separate chambers of a microfluidic device that allowed only axonal connections between compartments. PFFs were added to the first chamber, and after two weeks, phosphorylated αSyn was detected in neurons in the second and third chambers—indicating trans-neuronal spread of pathology [128]. In vitro, both full-length and truncated recombinant αSyn, when introduced into mouse hippocampal neurons, can induce synaptic dysfunction and neuronal death—even in the absence of detectable phosphorylated αSyn accumulation. This suggests that early toxic species may exert pathogenic effects before overt fibrillar deposition [129]. Furthermore, in vivo studies using brain homogenates from elderly patients carrying the A53T αSyn mutation have shown that intracerebral inoculation into young transgenic (TgM83) mice leads to widespread αSyn pathology in host neurons, early-onset motor symptoms, and neurodegeneration. Notably, this pathological spread can be suppressed by genetic deletion of endogenous αSyn in the host, underscoring the requirement of native αSyn for templated aggregation [130].

The PFF model has become a cornerstone in studying αSyn pathology and its prion-like propagation. In this model, synthetic αSyn fibrils are injected into specific brain regions to seed endogenous αSyn aggregation. For example, intra-striatal injection of αSyn PFFs into non-transgenic mice leads to the formation of perinuclear inclusions that mature into LB-like structures within approximately 90 days. These aggregates spread ipsilaterally through connected brain regions. Notably, no pathology is observed in αSyn knockout mice, confirming the necessity of endogenous αSyn for templated aggregation. The pathology is significantly more severe in transgenic mice expressing mutant αSyn, such as A53T [121]. In non-human primate models, injection of αSyn PFFs into the putamen has been shown to induce pathology in the SN, demonstrating the model’s relevance to human PD anatomy and progression [131]. More recently, researchers have used amplified αSyn aggregates derived from patients with LB disease. These patient-derived aggregates, when injected into mice expressing human αSyn, produce pathology with a proteinase K digestion pattern that closely resembles human brain lysates—distinct from the pattern seen with synthetic PFFs [123]. This suggests that amplified aggregates may better mimic the biochemical and structural features of human LB than synthetic fibrils.

Multiple lines of evidence support αSyn being a prion-like protein. αSyn oligomers can be amplified from multiple tissues sources in vitro using seed amplification assays (SAAs) [132]. These assays, adapted from prion disease diagnostics, have now been translated into clinical practice as highly sensitive and specific tests for PD, particularly using CSF as the seed source. Recent large-scale studies, such as those from the PPMI, report SAA sensitivity of approximately 88–97% and specificity exceeding 96%, with the ability to detect pathological αSyn even in prodromal individuals before motor symptom onset [133].

αSyn “seeds” derived from recombinant proteins or lysates of diseased brains can propagate in rodent models of PD through intracerebral or peripheral induction [134]. Transmission of αSyn does not occur in SNCA knockout mice [135]. The Braak model describes the spatiotemporal progression of Lewy pathology [136]. Additionally, Lewy pathology can spread from host neurons to grafted neurons [137]. Interestingly, truncal vagotomy has been shown to reduce the risk of developing PD by 40–50% within five years after the procedure [138]. However, there are also some factors that are not well explained by the prion-like theory. There is no evidence of intraspecies or interspecies transmission of αSyn [139]. Grafted neurons in PD remain functional for 1 to 2 decades post-transplantation [140]. Lewy pathology exhibits a patchy distribution that does not fully align with the expected connectome [141]. Additionally, clinical progression does not always correspond consistently with pathological findings. Furthermore, αSyn pathology has been detected in multiple peripheral organs—including the gastrointestinal tract, salivary glands, and skin—without a clear temporal sequence or connectivity pattern, challenging the notion of a strictly linear propagation model [142].

#### 3.1.1. Structural Differences Among Different Synucleinopathies

There are structural differences that have been identified among different synucleinopathies, called strains. αSyn strains are conformations that retain specific, unique biochemical properties [143]. Also, αSyn strains have different biological effects in experimental models. One of the first studies that used aggregates from PMCA technique extracted them out from the CSF and then analyzed them. In the protofilaments and in the filaments generated from their extracts, patients with MSA compared to PD have more twists in these αSyn extracts [144]. And, the injection of variant concentrations in different cell line reveals that MSA lysate is much more toxic than in PD [145]. More recently, amplified extract aggregates from LB dementia showed that either single or double protofilament have very low twist [146].

Many studies in the last three to five years suggest that αSyn strains target different brain regions and cells. For example, the use of recombinant αSyn mutant with changed morphology by addition of sodium chloride showed different thresholds for thioflavin T fluorescence. The injection of both together with extracts from homogenous mutant mice brain show that non-soft fibrils and the homogenous mutant extracts have much more pathology and predilection for targeting astrocytes [147].

Recent work highlights that αSyn strain heterogeneity may underlie clinical differences between PD, DLB, and MSA, as well as variability within each disorder. Plasma-based studies using conformation-selective antibodies for two in vitro–derived αSyn strains (strain A and strain B) demonstrated that PD patients exhibit higher plasma levels of both strains compared to DLB, despite similar brain pathology at autopsy [75]. Notably, lower plasma strain A levels predicted faster cognitive decline in PD, suggesting that strain composition may influence cognitive trajectory. Furthermore, these plasma species were capable of seeding αSyn fibrillization in SAAs and inducing intracellular inclusions in cell models, supporting their pathogenic potential. Interestingly, these strain-specific differences were not reflected in CSF, implying a possible peripheral origin or compartmentalization of certain αSyn species [148].

#### 3.1.2. αSyn Aggregates from Periphery to Brain

Gut injection of pathologic αSyn causes PD-like motor and non-motor symptoms. PD-like pathology and symptoms require endogenous αSyn [149]. The injection of exogenous PFF of αSyn in the duodenum of normal mice produce both progression of pathology up to the brain and also clinical phenotype in the mouse. However, the cutting of the vagus nerve or injection of PFF of αSyn into the knockout mouse for SNCA may prevent the development of the disease. In three months, DMV are affected; and, after 7–10 months, there are lesions in the striatum and locus coeruleus. Also, loss of TH stain can be observed in 10 months with loss of dopamine transporter signals [150]. A mouse model of gut-induced αSyn and tau co-pathology demonstrates the ability to spread into the brain. In the early stages, pathology originating in the gut propagates to the DMV and the nucleus tractus solitarius (NTS). Behavioral impairments related to anxiety, cognition, and motor function emerge progressively over time. Truncal vagotomy and αSyn deficiency have been shown to inhibit the spread of synucleinopathy and tauopathy [151].

There is evidence for the interaction between the gut microbiome, intestinal health, and PD-related neurodegeneration. The presence of human αSyn in the midgut is linked to a shortened lifespan, loss of dopaminergic neurons, and progressive motor defects. Age-related changes contribute to the onset of intestinal barrier dysfunction, tight junction disruption, and dysbiosis, leading to an increased bacterial load, including elevated levels of Proteobacteria. Microbiome depletion has proven effective in restoring intestinal homeostasis and mitigating the progression of PD symptoms in animal models [152].

**Figure 3:**
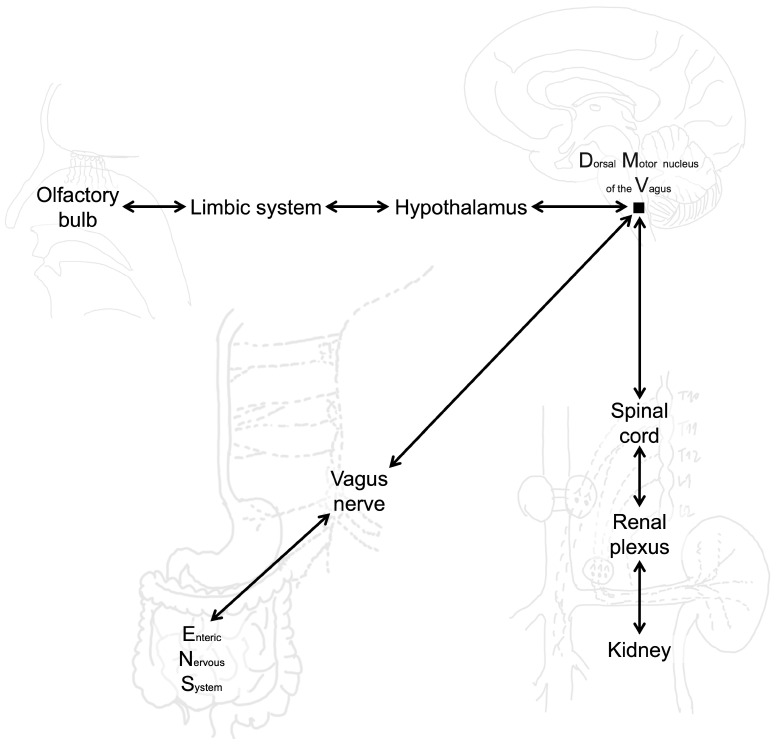
Schematic representation of proposed αSyn propagation pathways from peripheral organs to the brain. Illustrated are three hypothesized routes by which misfolded αSyn may ascend from peripheral sites to central nervous system structures. Nasal–brain axis: αSyn enters via the olfactory epithelium, propagates through the olfactory bulb and limbic system, and may reach brainstem autonomic centers via multisynaptic connections through the hypothalamus. Kidney–brain axis: aggregates may travel via sympathetic fibers originating in the renal plexus, ascending through the spinal cord to brainstem nuclei. Gut–brain axis: αSyn spreads from the enteric nervous system via the vagus nerve to the dorsal motor nucleus of the vagus (DMV). Arrows indicate proposed directions of propagation.

Experimental and pathological studies indicate that αSyn can spread to the CNS through multiple peripheral routes beyond the gut–brain axis. The kidney functions as both a clearance organ and a potential reservoir for pathogenic αSyn. Impaired renal function reduces αSyn degradation, leading to its accumulation in renal tissue and subsequent propagation to the CNS via renal sympathetic and parasympathetic innervation. In mouse models, intrarenal injection of αSyn PFFs triggered widespread CNS pathology—including SN and cortical regions—dopaminergic neurodegeneration, and motor deficits, all of which were prevented by renal denervation, confirming a kidney–brain neuronal transmission pathway [153]. Similarly, the nasal mucosa represents another entry point for αSyn. A single intranasal administration of αSyn PFFs in wild-type mice induced Lewy neurite-like aggregates in the olfactory bulb, supporting the hypothesis that olfactory structures may serve as an initial site for αSyn entry into the brain. Although propagation beyond the olfactory bulb was limited in this short-term model, these findings underscore the vulnerability of the olfactory route, particularly under conditions that increase mucosal permeability [154]. Collectively, these observations suggest that multiple peripheral gateways—including kidney and nasal pathways—may contribute to the initiation and progression of synucleinopathies, with implications for disease heterogeneity and therapeutic targeting (Figure 3). However, the precise contribution of peripheral mechanisms to the generation and propagation of αSyn pathology remain uncertain.

### 3.2. αSyn as a Biomarker for PD

αSyn pathology is detected in up to 8.3% of elderly individuals without clinical symptoms, indicating that its presence alone is not sufficient for PD manifestation. In AD, LB pathology is observed in approximately 31.5% of cases—a notable proportion given that AD is primarily defined by Aβ and tau pathology [155]. This co-occurrence suggests mechanistic overlap between neurodegenerative processes and may contribute to clinical heterogeneity, including accelerated cognitive decline.

There are reports in the literature of PD with homozygous exon deletion in parkin with severe neuronal loss in the SN without any LB [156], also similar findings were observed in autopsy of patients with LRRK2 p.R1441H homozygous and heterozygous mutations [157]. Moreover, no correlation of nigral neurodegeneration with LB distribution or density has been found. Region specific pattern of αSyn aggregation is not parallel to regional patterns of cell death. The demise of nigral neurons/loss of dopa markers precede nigral αSyn aggregates. Lewy pathology is not the first sign of degeneration in vulnerable neurons. Hippocampal and motor cortex, may also display neuronal loss with minimal LB-related pathologies [158]. More recently, in the PPMI study it was found that LRRK2 parkinsonism cases without αSyn aggregates have less severe motor but more cognitive dysfunction suggesting that other mechanisms other than αSyn aggregation may be more prominent in the pathophysiological mechanism of PD [159].

One of the major advances is that αSyn can be extracted from extracellular compartments such as CSF, blood, saliva, skin, and digestive tract. Also, specific αSyn types can be targeted with the evolution of the detection methods including immunoassays (moderately specific, moderately sensitive), mass spectroscopy (multiplex detection, less sensitive), and SAAs (sensitive but maybe less specific) [160].

Advancements in detection methods, particularly SAAs such as RT-QuIC and PMCA, have significantly enhanced the ability to detect misfolded αSyn aggregates with high sensitivity and specificity, even at very low concentrations [161,162]. These assays have been successfully applied to a range of biological matrices, including CSF, blood, skin, ECV, saliva, OM, GIT, and SMG [163]. A recent meta-analysis reported overall sensitivity of 86% and specificity of 92%, with CSF, skin, blood, and ECVs demonstrating the highest diagnostic accuracy [164].

Among non-invasive sources, tear fluid has shown promise as a biomarker, with αSyn levels differentiating PD patients from controls, although further studies are needed to establish its correlation with disease progression [165]. Skin biopsy is also emerging as a valuable tool, enabling immunohistochemical detection of phosphorylated αSyn in peripheral autonomic nerves. Sampling from regions such as the cervical region and distal leg have shown potential for distinguishing PD from atypical parkinsonian syndromes [166]. Its minimally invasive nature and potential for differentiating synucleinopathies make it a compelling option for clinical application. Notably, ECVs and blood are gaining priority due to their high diagnostic performance and accessibility, while GIT, OM, and oral samples show limited sensitivity. Saliva and SMG require further methodological refinement to reach clinical utility [164].

## 4. Different αSyn Strains in PD Brains

In PD, αSyn is deposited in LB. However, it is unclear if there are strain differences from one patient to other and if these differences might modulate the progression of PD (Table 4) [167,168,169,170]. A biological strain is a specific biochemical composition of a molecule that retains unique phenotypic or genotypic traits.

Yang et al. purified certain types of aggregates with N-lauroylsarcosine of PWP, PDD, and DLB. They noticed the same arrangements of αSyn in the aggregates in the top-view of cryo-EM with atomic resolution varying from 2.2 to 3.5 Å [171]. EM studies capture only a subset of the total population of αSyn fibrils in specific views so this method may not be sufficient to detect minor strain-specific differences. Another analysis performed by the same group in patients diagnosed with MSA and juvenile-onset synucleinopathy demonstrated significant difference between PD and MSA [172] which may be due to genetic variations or different αSyn strains.

Further evidence of different types of αSyn strains in PD is different types of staining. A study comparing patients with MSA and PD revealed different fluorescence properties between these two diseases, showing that dual labelling with spectral analysis and phosphorylation of serine-129 can different both pathologies (MSA appears red and PD appears blue). Also, the authors observed two types of PD one with fluorescence similar to MSA (low 486/573R wave length) and another completely different [173].

The brain tissue of patients with MSA and PD were added to seeds to amplify the results of αSyn aggregation. After this, the authors used hydrogen/deuterium exchange to study the protein structure and dynamics. The results were plotted in graphs with protonation (accessibility and flexibility of αSyn) and residue number. Interestingly, there was visible difference between the 20–50 residue numbers between these two neurodegenerative conditions (PD with lower protonation and MSA with high protonation) [174]. Moreover, there is notable variability in the internal molecular structure among individuals with PD or MSA and lower heterogeneity in MSA when compared to PD [144]. This was further supported by SAAs of αSyn from the CSF with decreased maximum amplification signal in samples from MSA relative to PD [144,175]. Also, the PPMI cohort revealed different fluorescence data across the spectrum of PD in patients with sporadic and genetic cases; but, it is unclear the association of this findings with the internal variation in αSyn structure [176].

Frieg et al. amplified αSyn seeds extracted from human PD and MSA brains using PMCA, then characterized the resulting fibrils by EM and immunostaining (p25α, D37A6, LB509). When applied to primary oligodendroglial cultures and injected into wild-type mouse brains, MSA-derived fibrils exhibited strong seeding and toxicity, whereas PD-derived fibrils showed minimal propagation. In the cryo-EM in PD, it was observed that the β2 structure interacts with β1 from MSA; also, most of the αSyn structure in PD is positively charged, and the internal interaction are negative. Therefore, the quaternary structure can be relevant for generation of different pathologies [175].

Genetics has an important role for not only genetic forms of PD but also influences the risk for the sporadic form. Knowledge is scarce regarding how the mutations in αSyn gene alter the internal molecular structure of αSyn deposits in the LB and impact development of various αSyn strains. All the mutations of αSyn gene known so far (A53T, A53E, A53V, H50Q, G51D, E46K, A30P, A30G, and V15A) are located in the curly region that interact with other membranes suggesting that the interaction of the protein with other membranes is important for the disease state [177]. A study using nuclear magnetic resonance spectroscopy assessing wild-type and V15A αSyn with liposomes aggregation revealed that in V15A cases the proteins have lower affinity between membranes with lower interaction resulting in less twisting [178].

Fibrils amplified from brain extracts of PWP and MSA exhibit distinct structural characteristics. PD-derived αSyn fibrils show greater structural diversity compared to those propagated from MSA brain extracts. The quaternary structure of αSyn fibrils plays a critical role in their neurotoxicity and capacity for propagation. These structural variations may correlate with specific PD subtypes, potentially supporting efforts in disease subtyping.

## 5. Co-Pathology in PD

### 5.1. Tau and αSyn

**Figure 4:**
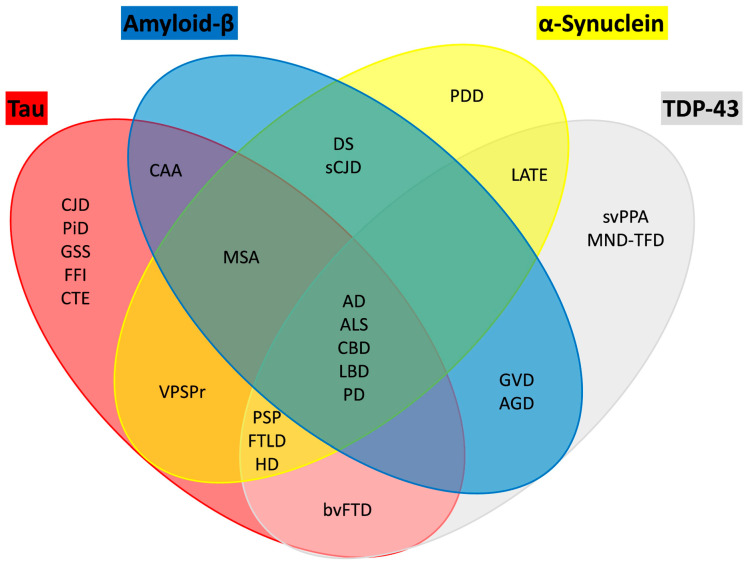
Tau, amyloid-β, αSyn, and TDP-43 proteinopathies. AD, Alzheimer’s disease; AGD, argyrophilic grain disease; ALS, amyotrophic lateral sclerosis; bvFTD, behavioral variant frontotemporal dementia; CAA, cerebral amyloid angiopathy; CBD, corticobasal degeneration; CJD, Creutzfeldt-Jakob disease; CTE, chronic traumatic encephalopathy; DS, Down syndrome; FFI, fatal familial insomnia; FTLD, frontotemporal lobar degeneration; GVD, granulovacuolar degeneration; GSS, Gerstmann-Sträussler-Scheinker; HD, Huntington’s disease; LATE, limbic-predominant age-related TDP-43 encephalopathy; LBD, Lewy body dementia; MND-TFD, frontotemporal dementia with motor neuron disease; MSA, multiple system atrophy; PD, Parkinson’s disease; PDD, Parkinson’s disease dementia; PiD, Pick’s disease; PSP, progressive supranuclear palsy; sCJD, sporadic Creutzfeldt-Jakob disease; svPPA, semantic variant primary progressive aphasia; VPSPr, variably protease-sensitive prionopathy.

PD is often associated with the presence of other neurodegeneration-associated proteins, termed co-pathology (Figure 4). In terms of the common co-pathologies, Aβ is found in 55% of the patients with PD and over 80% of the patients with LB dementia. Also, hyperphosphorylation of tau is observed in over 50% of the individuals with both PD and LB dementia [179]. Therefore, classifying individuals based solely on αSyn pathology may not capture the full spectrum of neurodegenerative processes. Most therapeutic trials for PD enroll patients within five years of diagnosis [180]. However, by this stage, the SN often shows near-complete loss of dopaminergic neurons, as indicated by the absence of TH staining in the putamen, suggesting that interventions targeting neuronal survival may be less effective. This underscores the need to identify and treat patients earlier in the disease course.

Biomarker development is critical for this goal. αSyn SAA have transformed the field by enabling detection of misfolded αSyn in peripheral tissues and fluids, offering a window into prodromal disease [181]. For example, longitudinal studies such as the Religious Orders Study and Memory and Aging Project (RUSH) demonstrated that individuals with parkinsonism—an intermediate state between healthy aging and PD—often harbor αSyn pathology before clinical diagnosis [182]. These findings align with neuropathological data showing that PD pathology begins years before motor symptoms emerge [180]. Incorporating such biomarkers into clinical trial design could allow enrollment of individuals at earlier stages, when dopaminergic neurons are more likely to be preserved and responsive to neuroprotective or restorative therapies [182].

In patients with only a clinical diagnosis of PD, the individuals were divided into non-motor impairment, mild-motor impairment without LB pathology, mild-motor impairment with LB pathology, and PD. In terms of striatal nigral cell loss, putamen innervation, AT8 staining, there was no difference between patients with motor impairment independently of the LB pathology. The authors also performed CP13 (earlier tau marker) and PHF (paired helical filaments) to confirm the results of AT8 staining. An important limitation of the study is that these patients lived a long-time and may not constitute what is in the normal population [183]. Interestingly, about 50% of the PWP and LRRK2 mutation may have LB pathology [184], and some of them with G2019S [185] and I2020T [186] may also have tau pathology. Also, there are cases of absence of LB pathology in patients with PINK1 homozygous mutations [187]. In this context, experimentally the knock-in VPS35 LRRK2 expressed only tau and no LB pathology with dopaminergic cell loss [188]. On the other hand, LRRK2 G2019S knock in mice revealed both tau and LB pathology [189]. Moreover, the first sign of degeneration is nigral degeneration before the occurrence of Lewy pathology [190].

Intra-AAVP320F/P301L mutant tau injected into the monkey entorhinal cortex with animal sacrificed three and six months later. These mutations rarely occur together in the same individuals, but the experimental model was performed to be a more aggressive disease and make it more minimal to reasonably time studies. The pathological flow of tau in these models is entorhinal cortex to hippocampus, followed by retrosplenial cortex, visual cortex, and later to the prefrontal cortex and temporal neocortex. The rhesus model of tau has decreased levels of BDNF and increased levels of neurofilament light chain, total tau, TDP-43 (transactive response DNA-binding protein 43), pTau S396, pTau S199, pTau T231, sTREM2 (soluble triggering receptor expressed on myeloid cells 2), TNF-α, IL-6, Aβ40, and Aβ42. Progression of pathology from pre-tangle AT8 staining to neurofibrillary tangle formation followed AAV-double mutant tau injections into the monkey entorhinal cortex. The authors also checked permissive templating with isoforms 4R being found into the entorhinal cortex in greater extent than 3R; and the 3R was mainly found in the hippocampus. Interestingly, in vivo tau PET tracers can be performed, so different therapies can be studied without sacrificing the animal. Also, in three months, the authors observed no cell loss and pre-tangles; and, in 6 months, cell loss and tangles were seen [191].

A murine model of co-pathology placed the vector into the entorhinal cortex of a J20 mice, which may overexpress Aβ. The authors observed increased inflammation which may be a driver of pathology. Cell loss was only observed in the co-pathology model. The vector was also assessed in the monkey model AT8 and PFFs into the striatum and aged animal that has naturally occurring Aβ. Also, the monkey models revealed the worst motor function in cases of co-pathology; moreover, the findings were only statistically significant in the first two months. Cell loss was mainly noticed in the co-pathology models, but in probands with only tau was only observed reduction in neurons. AT8 expression following nigral injection of AAV-double mutant tau is robust and has amazing properties of propagation in aged monkeys [192].

In sum, tau and Aβ frequently co-occur with αSyn pathology in PD and related disorders, and their presence is associated with more severe neurodegeneration and cognitive decline [193]. Autopsy studies indicate that Aβ plaques are present in over half of PD cases and in the majority of patients with LB dementia, while hyperphosphorylated tau is observed in a similar proportion [194]. These additional pathologies accelerate disease progression, contribute to earlier onset of dementia, and increase cortical involvement compared to cases with αSyn pathology alone.

### 5.2. LB Disease and AD

LB disorders, including PD and DLB, frequently exhibit coexisting AD pathology, characterized by Aβ plaques and tau neurofibrillary tangles. This overlap is not incidental; it reflects a mixed proteinopathy that significantly influences clinical outcomes. Studies show that Aβ and tau burden in LB disease correlates with accelerated cognitive decline, earlier onset of dementia, and increased mortality compared to cases with αSyn pathology alone [195]. Mechanistically, Aβ and tau may exacerbate αSyn aggregation through cross-seeding and synergistic toxicity, amplifying neurodegeneration.

Up to 50% of LB disease patients have AD pathology, and AD is related with a more severe disease course (high risk of nursing home admission and death) in LB disease [196]. In LB disease patients, various cognitive domains are affected, such as executive function, memory, and visuospatial function. Frontal executive impairment is more commonly associated with LB pathology, and no association with earlier dementia and even improvement in some patients was observed [197]. On the other hand, posterior cortical impairments in memory and visuospatial function are more common related with AD co-comorbidity and progressed more rapidly to dementia [198].

Pathophysiological processes underlying the coexistence of AD in LB disease, remain unknown, and blood biomarkers, which can identify a comorbid AD pathology have not yet been established [199]. Plasma Aβ biomarkers using Simoa^®^ assay were less accurate in detecting Aβ pathology in LB disease and there was no difference between Aβ40/Aβ42 ratio in positive or negative PET Aβ [200]. Nevertheless, plasma Aβ-biomarkers using immunoprecipitation-mass spectrometry (IP-MS) predict brain Aβ burden at an individual level in patients with AD [201]. The authors found significant results with Aβ40/Aβ42 ratio in differentiating patients with LB disease without Aβ, LB disease with Aβ, and AD with sensitivity of 0.6, specificity 0.6, and area under the curve of 0.68 [202]. Therefore, the plasma Aβ40/Aβ42 ratio was significantly decreased in Aβ positive LB disease patients compared to those without Aβ, suggesting that plasma Aβ biomarkers are useful to detect the presence of Aβ pathology coexisting with LB disease.

There are reports of cross-seeding effects of Aβ and αSyn [203]. The αSyn aggregates from LB disease patients with comorbid AD pathology were highly toxic to neurons particularly the Apolipoprotein E (APOE) 34 allele carriers [204].

## 6. Therapeutic Targets

Advances in molecular biology and pharmacology have enabled the development of agents that modulate immune responses or directly interfere with disease-driving proteins. Among these, small molecules and biologics aimed at αSyn, tau, and Aβ have gained attention for their potential to alter disease progression and improve clinical outcomes.

### 6.1. Ongoing Clinical Trials

The clinical trial NCT05357989 evaluated the efficacy and safety of buntanetap in PWP. Buntanetap is a small molecule that inhibits neuro-toxic protein aggregates such as αSyn, Aβ, and tau. This Phase 3 study enrolled 450 participants aged 40–85 years, who were randomized to receive either low-dose (10 mg), high-dose (20 mg) buntanetap, or placebo over a three-year period. The results demonstrated that patients in the 20 mg buntanetap group exhibited significant improvements in motor and non-motor functions, as measured by the Movement Disorder Society-Unified Parkinson’s Disease Rating Scale (MDS-UPDRS) Parts II and III, compared to the placebo group. Additionally, cognitive function, assessed using the Mini-Mental State Exam (MMSE), was preserved or improved in the buntanetap groups, whereas the placebo group experienced cognitive decline [205].

Another notable study, NCT04777331, known as the PADOVA trial, is a Phase 2b, multicenter, randomized, double-blind, placebo-controlled study assessing the efficacy and safety of intravenous prasinezumab in participants with early PD. Prasinezumab is a monoclonal antibody targeting aggregated αSyn [206]. Although the trial did not meet its primary endpoints, a post hoc subgroup analysis revealed that prasinezumab may slow motor progression in patients with rapidly progressing disease, as measured by MDS-UPDRS Part III scores. This subgroup exhibited more homogeneous progression rates, which reduced data variability and increased the statistical power to detect treatment effects. However, Xiao et al. caution that these findings should be interpreted with tempered optimism due to the retrospective nature of the analysis and potential confounding factors [207].

In the realm of immunotherapy, the clinical trial NCT05634876 is a Phase 1b study designed to evaluate the safety, tolerability, and immunogenicity of UB-312 in participants with MSA. UB-312 is an investigational vaccine intended to elicit an immune response against toxic forms of αSyn. Preliminary findings from a Phase 1 trial indicated that UB-312 effectively reduced damaging clumps of αSyn in treated patients [208].

Another investigational approach involves MEDI1341, a human IgG1λ monoclonal antibody engineered for selective, high-affinity binding to αSyn with reduced effector function. This therapeutic strategy aims to mitigate the pathological aggregation of αSyn in the CNS, thereby potentially altering the course of PD [209].

UCB0599 (minzasolmin) represents a distinct therapeutic approach in PD aimed at modulating αSyn pathology through small-molecule inhibition. It is an orally administered compound is designed to prevent the misfolding and aggregation of native αSyn [210]. The drug advanced to the Phase 2 ORCHESTRA trial, which evaluated its safety, tolerability, and potential disease-modifying effects in individuals with early-stage PD (NCT05543252). Despite a strong mechanistic rationale, the study failed to demonstrate clinical efficacy on motor and functional outcomes, and its long-term extension was subsequently terminated in 2025 (https://www.ucb.com/newsroom/press-releases/article/findings-from-minzasolmin-proof-of-concept-orchestra-study-shape-next-steps-in-ucb-parkinson-s-research-program, accessed on 23 October 2025).

Collectively, these clinical trials underscore the ongoing efforts to develop disease-modifying therapies targeting αSyn in PD and related disorders. The pursuit of immunotherapeutic strategies, including monoclonal antibodies and vaccines, reflects a concerted effort to address the underlying pathophysiology of these neurodegenerative diseases. However, the idea of mainly targeting only one pathway can lead to significant misunderstanding of the pathologies related to PD. There is evidence of tau and αSyn pathologies in different individuals. For further comprehension of the clinical trials about immunotherapy targeting αSyn in PD, consider reading Table 5.

#### 6.1.1. Rationale for Dual Targeting of αSyn and Tau in PD

Given mounting evidence that αSyn and tau pathologies interact in PD, combination strategies are increasingly rational. Autopsy series indicate that, while neocortical LB pathology is the principal substrate of dementia, one-third of PDD cases exhibit moderate–severe tau and half have moderate–severe Aβ, and higher tau/Aβ burdens track faster cognitive decline and mortality, suggesting that concurrent tau targeting could enhance disease modification in cognitively vulnerable PD populations [211]. Experimental work demonstrates biological synergy, with tau accelerating αSyn aggregation and spread and reviews summarizing cross-seeding across synucleinopathies and tauopathies [212]. Genetic studies further implicate tau in PD pathogenesis, with the MAPT H1 haplotype associated with increased disease risk and altered tau isoform expression [213]. These findings support the concept of combination therapies that address both αSyn and tau to slow disease progression. Clinically, αSyn immunotherapies have produced mixed outcomes: prasinezumab did not meet its primary endpoint in the PASADENA trial, yet showed signs of motor benefit and a sustained effect in the open-label extension, suggesting partial efficacy in slowing disease progression [214]. In contrast, cinpanemab (BIIB054) failed to demonstrate any clinical benefit in the SPARK trial [215], highlighting the limitations of targeting extracellular αSyn alone and underscoring the need to also reduce intracellular αSyn pools through complementary approaches such as antisense oligonucleotides (ASO) or gene therapy.

#### 6.1.2. Antisense Oligonucleotides

SNCA-targeted ASOs offer a promising strategy to overcome the intracellular limitations of prior αSyn-directed therapies. Preclinical studies demonstrate prevention and reversal of αSyn pathology with broad brain distribution and CSF target engagement in non-human primates—while early human experience with SNCA-ASOs in synucleinopathies supports safety and pharmacodynamics (NCT03976349). In parallel, tau-lowering with intrathecal ASOs (e.g., BIIB080) has reduced CSF tau and tau-PET in humans, providing a translatable platform for PD trials enriched for tau co-pathology (e.g., MAPT H1/H1 carriers, elevated plasma pTau217) (NCT05399888). BIIB101 was another ASO targeting αSyn initially developed for MSA but reflected broader hopes of extending such approaches to PD. Although BIIB101 progressed to a Phase 1 safety and pharmacokinetic study, its development was discontinued in early 2025 due to limited progress (NCT04165486).

Combination therapies targeting both αSyn and tau may offer synergistic benefits in PD, especially in patients with mixed motor and cognitive phenotypes. One proposed strategy involves pairing an extracellular αSyn antibody or vaccine with an intracellular SNCA-lowering agent, such as an ASO or gene therapy [216].

#### 6.1.3. Zinc Finger Repressors

Preclinical studies demonstrated that AAV-packaged zinc finger repressors (ZFRs) achieved robust and specific repression of αSyn in human neuronal cell lines and in vivo in a humanized mouse model. Following tail vein injection of AAV-ZFR, significant SNCA knockdown was observed in dopaminergic neurons of the SN and thalamus, regions critically affected in PD [217]. This approach offers a potential one-time therapy that directly reduces intracellular αSyn levels, complementing extracellular immunotherapies and supporting combination strategies aimed at halting disease progression.

### 6.2. Exercise

Exercise slows the progression of PD, but the mechanism of this association is poorly understood. One possibility is a protein called irisin, which is a hormone-like protein that regulates energy expenditure, insulin use, and inflammation [218]. It is secreted from skeletal muscle during exercise, and it has been shown in mice and humans that exercise irisin is secreted into the bloodstream, and it readily crosses the blood-brain-barrier. Irisin improves neurodegenerative phenotypes through a wide array of mechanisms, including the clearance of misfolded and aberrantly phosphorylated proteins (Aβ, tau, and αSyn), induction of autophagy, upregulation of BDNF expression, and reduction in inflammation, among other things [219]. Irisin-flag levels will be measured in plasma using ELISA, with the aim of establishing a clinically relevant biomarker assay in a non-human primate model. This represents the first disease-modifying therapeutic capable of addressing multiple pathologic proteins associated with neurodegenerative diseases, as well as both motor and non-motor symptoms, through a single intravenous dose (https://www.cureparkinsonsnz.org.nz/keeping_abreast_of_global_progress/, accessed on 23 October 2025).

While irisin is a compelling candidate, exercise-induced neuroprotection in PD likely arises from a constellation of molecular mechanisms. These include upregulation of neurotrophic factors such as BDNF, GDNF, and CDNF, which promote neuronal survival and synaptic plasticity [220]; enhancement of mitochondrial biogenesis and turnover via AMPK–PGC-1α signaling, improving energy metabolism and reducing mitochondrial dysfunction [221]; increased antioxidant capacity, with elevated enzymes like MnSOD, GPX4, and catalase mitigating oxidative and nitrosative stress [222]; attenuation of endoplasmic reticulum stress and restoration of calcium homeostasis through SERCA2 and CaMKIIα regulation [223]; and activation of autophagy and mitophagy, facilitating clearance of misfolded proteins and damaged organelles [224]. Notably, high-intensity aerobic exercise has been shown to restore dopaminergic neuron integrity, improve synaptic connectivity, and elevate dopamine release in the striatum [225]. These multifaceted effects suggest that exercise acts as a systems-level intervention, engaging diverse cellular processes to counteract neurodegeneration.

### 6.3. Cell-Surface Receptors Mediating αSyn Uptake

The prion-like propagation of misfolded αSyn is increasingly recognized as an active process involving specific cell-surface receptors that facilitate extracellular αSyn binding, internalization, and subsequent intracellular signaling.

Low-density lipoprotein receptor-related protein 1 (LRP1) has emerged as a key neuronal receptor for αSyn uptake. Genetic deletion of *Lrp1* in neurons significantly reduces internalization of monomeric and oligomeric αSyn and attenuates its spread in mouse hippocampus. Uptake depends on lysine residues and the N-terminal domain of αSyn, suggesting a defined interaction interface. While no clinical inhibitors exist, preclinical strategies include competitive ligands and lysine-targeted chemistries to block LRP1-mediated uptake [226].

Heparan sulfate proteoglycans (HSPGs) act as glycan-based receptors for αSyn fibrils. CRISPR screens and in vivo models confirm that sulfation patterns of heparan sulfate critically regulate fibril binding and endocytosis. Pharmacologic approaches such as heparin mimetics and sulfation inhibitors reduce αSyn uptake in cell systems, though translation to PD models remains early-stage [227].

Microglial TLR2 recognizes oligomeric αSyn as a damage-associated ligand, triggering NF-κB activation and inflammasome signaling. Similarly, TLR4 mediates glial inflammatory responses to αSyn aggregates. Neutralizing antibodies and small-molecule TLR modulators have shown efficacy in reducing neuroinflammation and αSyn pathology in rodent models, positioning innate immune receptors as promising targets [228].

Initial studies implicated lymphocyte-activation gene 3 (LAG3) as a receptor for αSyn fibrils, with *Lag3* knockout reducing pathology in A53T mice [229]. Recent work suggests that LAG3 cooperates with amyloid precursor-like protein 1 (APLP1) to facilitate fibril uptake, though controversy persists regarding neuronal expression and specificity [230]. The availability of anti-LAG3 antibodies (e.g., relatlimab) in oncology raises the possibility of repurposing for PD, but clinical validation is lacking [231].

β-Neurexins [232] and sortilin [233] have been identified as additional neuronal receptors for αSyn fibrils, while purinergic P2X7 receptors may contribute to inflammatory signaling [234]. These findings broaden the receptor landscape but remain at a preclinical stage. Targeting receptor-mediated uptake offers a rational strategy to slow αSyn propagation. However, given receptor pleiotropy and cell-type specificity, selective inhibition without disrupting essential physiological functions remains a major challenge.

## 7. αSyn and Tau Radiotracers

Advances in PET tracers targeting αSyn have opened new possibilities for imaging synucleinopathies. The fluorinated ligand [18F]ACI-12589 demonstrated strong binding in MSA, allowing clear differentiation from healthy controls, but its sensitivity in PD and DLB remains limited due to the relatively low density of LB pathology [235]. To address this limitation, compounds such as [18F]C05-05 have been developed [236], enabling visualization of αSyn aggregates in the midbrain of individuals with PD and DLB and currently undergoing evaluation in the PPMI [237]. Other candidates, including the MODAG series ([11C]MODAG-001/-005) [238] and Merck’s MK-7337, have progressed into early human studies, though issues such as off-target binding and selectivity over tau and Aβ remain (IRAS ID: 334626). Novel chemotypes like M503-1619, which shows high affinity for αSyn fibrils (K_D = 2.5 nM) and minimal cross-reactivity with Aβ, represent promising directions for future clinical translation [239].

Although PD is primarily characterized by αSyn pathology, tau accumulation plays a significant role in cognitive impairment, particularly in PDD and PD with mild cognitive impairment (PD-MCI). PET tracers such as [18F]AV-1451, [18F]THK-5351, and second-generation ligands like [18F]PI-2620 have been used to map tau deposition patterns. A meta-analysis of 15 studies reported higher tau tracer uptake in the inferior temporal and entorhinal cortices of PDD and PD-MCI patients compared to healthy controls, while uptake in PD without dementia was lower and more variable [240]. Compared with AD and progressive supranuclear palsy, tau burden in PD is generally lower and more localized, but its presence correlates with cognitive decline and disease progression. The PPMI NeuroEXPLORER study is currently applying high-resolution PET with [18F]PI-2620 to quantify tau pathology in sporadic and LRRK2 PD, aiming to clarify its role in disease heterogeneity and its potential as a biomarker for clinical trials [241].

Tracer development for PD faces multiple obstacles. For αSyn imaging, the low abundance of pathological aggregates in early PD and structural heterogeneity across synucleinopathies complicate ligand design and sensitivity requirements [242]. Selectivity remains a critical issue, as co-pathologies involving tau and Aβ are common in PD, requiring tracers with minimal cross-reactivity [243]. For tau imaging, second-generation tracers such as [18F]PI-2620 have improved specificity and reduced off-target binding, but longitudinal studies are needed to validate their prognostic value and utility in monitoring therapeutic response. Structure-based ligand design informed by cryo-EM data and the development of new chemotypes such as arylpyrazolethiazoles for αSyn may further enhance brain penetration and binding affinity [244]. These strategies aim to integrate molecular imaging into precision medicine approaches for PD diagnosis, progression tracking, and treatment evaluation.

## 8. Future Directions

In summary, current evidence suggests that the elongation and aggregation of pathologic αSyn which then spreads and induces misfolding of normal αSyn is a significant pathologic mechanism in multiple neurodegenerative diseases. Possible therapeutic strategies may involve blocking the release of misfolded αSyn, blocking uptake by neighboring cells of αSyn, preventing seeding from normal to abnormal conformational change, and promoting degradation of the misfolded αSyn.

Experimental evidence suggests that genetic mutations, protein modifications, protein interactions, and microenvironmental factors may regulate the conversion of monomers, oligomers, or fibrils. However, the impact of aging, comorbidities, drug effects, and lifestyle cannot be fully addressed through isolated laboratory experiments, where controlling variables are inherently limited. Therefore, it is essential to develop advanced methods to quantify specific protein species, track their intracellular and extracellular dynamics, and improve antibody targeting—especially for interaction sites that are currently inaccessible. Additionally, defining toxicity thresholds and understanding the influence of aging remain critical challenges.

While structural biology has significantly advanced in characterizing various polymer forms, the key question now is how to apply this knowledge to better understand clinical outcome progression, heterogeneity, and pathology. Detection of αSyn alone may be insufficient, highlighting the need for multiplex assays to monitor multiple proteins simultaneously. Furthermore, the development of precise imaging techniques for micro αSyn remains a priority to advance research and therapeutic strategies.

Recent studies using the C05-05 tracer for αSyn detection demonstrate both its potential and limitations [243]. The tracer effectively binds to αSyn in mouse models and postmortem brains affected by αSyn. However, in patients with MSA, it shows signal activation in the putamen and middle cerebellar peduncle [245]. Additionally, its affinity for tangles and plaques in patients with AD results in significant overlap between case and control groups, raising concerns about its specificity. Issues such as rapid metabolism, binding affinity, and in vivo stability further complicate its clinical utility.

Conceptually, strategies such as suppressing transcription, reducing translation, and increasing protein turnover are logical and well-understood. However, significant knowledge gaps remain. For instance, targeting extracellular αSyn has shown promise in recent computational and clinical trials, but critical challenges persist. Temporal changes in extracellular αSyn levels, dose-dependent toxicity thresholds, cell-to-cell transmission rates, receptor involvement, regulatory factors, and compensatory mechanisms require further exploration to better monitor disease progression over time.

Additionally, many existing antibodies fail to differentiate between distinct αSyn species or fragments of varying lengths. Their limited blood–brain barrier penetration and suboptimal distribution to specific brain regions further complicate therapeutic effectiveness. Moreover, if αSyn undergoes C-terminal truncation, antibodies targeting the C-terminal region may lose efficacy. Addressing these challenges is essential for guiding target selection and improving therapeutic engagement strategies.

Additional questions arise about how biological elements can be used to guide target selection, particularly in understanding cellular mechanisms, the spread of αSyn between cells, and the roles of various identified proteins. Insights into these processes are essential for refining therapeutic strategies and improving disease-modifying interventions.

## 9. Conclusions

αSyn is implicated in both monogenic and idiopathic forms of PD and is central to LB pathology, as supported by clinical and genetic studies. While animal models of αSyn capture certain features of clinical PD, they do not fully replicate disease complexity. The propagation of αSyn aggregates, their conformational species, distinct transmission trajectories, and regulatory factors remain poorly understood.

αSyn impacts multiple cellular functions and organelles, and neurodegeneration can occur even in the absence of detectable αSyn aggregates, suggesting the involvement of other proteins. The extracellular secretion of αSyn holds potential as a biomarker, particularly through seeding assays. However, no validated methods currently exist for direct in vivo tracking. Continued biological discoveries are essential to improve diagnostics, disease monitoring, and the development of effective therapeutic strategies.

In subjects with mild motor features, nigral cell loss, putaminal denervation, and dopamine phenotypic downregulation occur independently of αSyn aggregation. All cases with mild motor features, regardless of αSyn presence, exhibit tau hyperphosphorylation. The AAV-double mutant tau model is a powerful tool for studying tau’s role in neurodegenerative diseases. When PFFs are combined with AAV double mutant tau in aged, Aβ -expressing monkeys, it results in greater functional deficits and nigrostriatal degeneration compared to either PFFs or AAV double mutant tau alone. Finally, further study into therapeutics that disrupt the pathway of pathologic αSyn remain a promising future direction. For example, the exercise-related trophic factor, irisin, has been shown to clear αSyn, tau, and Aβ, suggesting its potential as a therapeutic agent for co-pathology diseases like PD and AD.

## Figures and Tables

**Table 1:** Allelic Variants of αSyn in PD.

Mutation	SNP	ClinVar	Clinical Phenotype	Levodopa Response	Disease Progression	Reference
Ala30Pro (A30P)	rs104893878	RCV000015045 (PD)	Mild dementia	Sustained response to levodopa	Same as typical PD	Krüger et al. (2001) [16]
Ala53Glu (A53E)	rs2116325; rs6842919	Merged with A53T	NA	NA	NA	Pasanen et al. (2017) [17]
Ala53Thr (A53T)	rs104893877	RCV000015044 (PD); RCV000526380 (PD & LBD); RCV004786261 (PD)	Asymmetrical bradykinesia, rigidity, dysautonomia, and rare head tremor	Good response to levodopa	Rapid	Athanassiadou et al. (1999) [18]
Glu46Lys (E64K)	rs104893875	RCV000015047 (LBD); RCV002514100 (PD)	Dementia and visual hallucinations	NA	Slightly faster than A30P based on assembly characteristics	Choi et al. (2004) [19]
Gly51Asp (G51D)	rs431905511	RCV000083251 (PD)	Dysautonomia, depression, anxiety, and cognitive impairment	Moderate response to levodopa	Rapid	Lesage et al. (2013) [20]
His50Gln (H50Q)	rs201106962	RCV000149507 (PD); RCV000344706 (PD); RCV001301465 (PD & LBD); RCV002307408 (NA); RCV002498683 (PD & LBD)	Cognitive impairment	Sustained response to levodopa	Slower	Kiely et al. (2015) [21]
Duplication	NA	RCV000015048 (PD); RCV000015049 (LBD)	Cognitive impairment and psychiatric decline	Sustained response to levodopa	Slower	Uchiyama et al. (2008) [22]
Triplication	NA	RCV000015046 (PD)	Cognitive impairment, psychiatric decline, and dysautonomia	Mild response to levodopa	Slower, more severe	Farrer et al. (2004) [23]

Abbreviations: LBD, Lewy body dementia; NA, not available/not applicable/unknown; PD, Parkinson’s disease; SNP, single nucleotide polymorphism.

**Table 2:** Effect on αSyn and post-translational modifications.

Post-Translation Modifications	Location	Effect on Aggregation	Reference
Acetylation	M1	⊖ aggregation rate of N-terminally acetylated αSyn than non-acetylated αSyn	Kang et al. (2012) [37]
O-GlcNAcylation	T72	⊖ aggregation, reduces PFF-induced toxicity	Marotta et al. (2015) [38]
	S87	⊖ aggregation: O-GlcNAcylated αSyn at S87 also inhibits the aggregation, but to a lesser extent than at T72	Lewis et al. (2017) [39]
	T75, T81	⊖ aggregation	Levine et al. (2019) [40]
	T33, T59, T64	Unknown	
Nitration	Y39	⊘: high MW nitrated forms inhibit aggregation; low MW forms promote it	Burai et al. (2015) [41] Qiao et al. (2019) [42]
	Y125	⊘: reduce aggregation or promotes dimerization	Burai et al. (2015) [41] Takahashi et al. (2002) [43]
	Y133, Y136	⊙: tyrosine-nitration blocks or does not affect αSyn fibril formation.	Norris et al. (2003) [44] Hodara et al. (2004) [45]
Phosphorylation	Y39	⨁ aggregation, especially in astrocytic αSyn	Brahmachari et al. (2016) [46]
	S87	⊖ aggregation	Paleologou et al. (2010) [47]
	S129	⨁ aggregation (dominant in LB), but there are studies showing decreased aggregation rate by enhancing autophagic degradation	Fujiwara et al. (2002) [48] Oueslati et al. (2013) [49]
	Y125, Y133, Y136	⊖ aggregation	Negro et al. (2002) [50]
SUMOylation	K96, K102	⊖ aggregation	Abeywardana et al. (2015) [51]
Truncation	C-terminal (e.g., 1–122, 1–119)	⊘: slow kinetics of αSyn propagation or enhance its assembly	Hoyer et al. (2004) [52] Serpell et al. (2000) [53]
	N-terminal	⊘: slows down or induce αSyn pathology	Terada et al. (2018) [54]
Ubiquitination	Lys residues (varied)	⊘: most ligases reduce aggregation (e.g., NEDD4, CHIP), others promote it (e.g., SIAH). It is more enzyme-dependent than residue.	Meier et al. (2012) [55] Rott et al. (2008) [56]

Abbreviations: MW, molecular weight. Notes: ⨁, promotes; ⊖, inhibits; ⊘, mixed results; ⊙, no effect.

**Table 3:** Pathological and Motor Phenotypic Characteristics of αSyn Mouse Models.

Feature	αSyn Tg	αSyn AAV Injection	αSyn PFF
Mechanism	Germline overexpression of human αSyn (WT or mutant, e.g., A53T) under neuronal promoters (e.g., Thy1, PrP)	Stereotaxic injection of AAV vector encoding human αSyn (WT or mutant) into SNpc or striatum	Injection of sonicated αSyn fibrils into brain (commonly striatum or SNpc)
Expression Level	Lifelong, supraphysiological (often >10× normal)	High, localized to injection site; depends on viral titer (e.g., 5.16 × 10^12^ gp/mL)	Normal endogenous αSyn misfolds and aggregates
Pathology Onset	Gradual, months (e.g., A53T Tg: motor signs at 8–12 months)	Rapid: Lewy-like pathology and TH+ neuron loss within 8–12 weeks	Progressive: inclusions appear ~1 month, neurodegeneration by 3–6 months
LB-like Inclusions	Present but often diffuse; variable morphology	Present in injected region; pS129 αSyn aggregates	Robust, widespread; mimics idiopathic PD with LB-like inclusions
Neurodegeneration	Variable; often incomplete nigrostriatal loss	Significant nigral DA neuron loss (e.g., 33% TH+ loss at 10 weeks)	Progressive nigrostriatal degeneration; circuit-specific vulnerability
Neuroinflammation	Chronic, due to overexpression	Localized to injection site	Follows inclusion formation; microgliosis and astrogliosis
Behavioral Deficits	Late-onset motor deficits (rotarod, grip strength)	Motor asymmetry detectable by 5–9 weeks (cylinder test)	Motor deficits after pathology develops (rotarod, wire hang)
Advantages	Stable, genetic PD model; reproducible	Flexible targeting; rapid onset; scalable	Mimics idiopathic PD; uses physiological αSyn levels; progressive
Limitations	Overexpression artifacts; long latency; non-physiological	Overexpression artifacts; limited spread beyond injection site	Requires surgery; slower than AAV for early readouts

Abbreviations: AAV, Adeno-associated virus; αSyn, Alpha-synuclein; DA, Dopaminergic; LB, Lewy body; PD, Parkinson’s disease; PFF, Preformed fibril; pS129 αSyn, Serine-129 phosphorylated alpha-synuclein; SNpc, Substantia nigra pars compacta; TH, Tyrosine hydroxylase; Tg, Transgenic; WT, Wild type.

**Table 4:** Structural features of αSyn strains.

Disease/Model	PD	MSA	DLB	Recombinant αSyn (In Vitro Type 1a)	Recombinant αSyn (In Vitro Type 2b)
RCSB.org Protein Data Bank ^a^	6SSX	6XYO	7L7H	6CU7	6CU8
Core residues ^b^	38–95	36–96	39–96	38–96	38–96
Protofilament arrangement ^c^	2 protofilaments, asymmetric	2 protofilaments, twisted	1 protofilament	2 protofilaments, parallel	2 protofilaments, antiparallel
Twist pitch ^d^	2160 Å	2160 Å	1728 Å	1728 Å	1728 Å
Resolution ^e^	2.98 Å	2.6 Å	4.0 Å	3.5 Å	3.5 Å
Distinctive features	Compact core, cavity absent	Large cavity, unique salt bridges	Extended β-sheet regions	Similar to PD strain but lacks PTMs	Distinct twist pitch, different interface contacts

^a^ Data obtained from https://www.rcsb.org/ accessed on 19 November 2025; Guerrero-Ferreira et al. (2018) [168]; Burger et al. (2021) [167]; Lee et al. (2022) [169]. ^b^ Core residues refer to the amino acid segment of the α-synuclein protein that forms the stable β-sheet-rich core of the fibril structure in each strain. ^c^ The way protofilaments associate to form a fibril: can be single (one protofilament) or paired (two protofilaments in parallel, antiparallel, or asymmetric configurations). ^d^ Twist pitch refers to the distance along the fibril axis required for the protofilament to complete one full helical turn, which can be calculated as “Pitch = Rise × (360°/Twist)” [170]. ^e^ Level of detail achieved in the Cryo-EM structure of the α-synuclein fibril. It is measured in Ångströms (Å).

**Table 5:** αSyn-Targeted Disease-Modifying Therapies for PD in Clinical Trials Registered in the ClinicalTrials.gov Database.

Study Start to Completion	Identifier	Intervention	N Enrolled	Comment
1 February 2012 to NA	NCT01568099	AFFITOPE® (AFFiRiS AG, Vienna, Austria) PD01A	32	Tolerability and safety of subcutaneous administration of two doses of AFFITOPE^®^ PD01A in early PD
1 June 2013 to NA	NCT01885494	AFFITOPE^®^ PD01A	30	AFF008E: observational phase 1b follow-up extension study for PWP after immunization with AFFITOPE^®^ PD01A
1 March 2014 to NA	NCT02095171	PRX002	40	Single ascending dose study of PRX002 in healthy subjects
1 June 2014 to NA	NCT02157714	PRX002	64	Multiple ascending dose study of PRX002 in PWP
1 December 2014 to 1 August 2016	NCT02267434	AFFITOPE^®^ PD03A	36	Study assessing tolerability and safety of AFFITOPE^®^ PD03A in patients with early PD (AFF011)
1 August 2014 to NA	NCT02216188	AFFITOPE^®^ PD01A	28	Follow-up study to assess one boost immunization with AFFITOPE^®^ PD01A with regard to safety and clinical activity (AFF008A)
1 July 2015 to 20 November 2017	NCT02459886	BIIB054	66	Single-ascending dose study of BIIB054 in healthy participants and early PD
2 February 2016 to 28 February 2017	NCT02618941	AFFITOPE^®^ PD01A	26	Follow-up study to assess a second boost immunization with AFFITOPE^®^ PD01A with regard to safety and clinical activity (AFF008AA)
5 August 2016	NCT02758730	AFFITOPE^®^ PD01A	0	Study assessing tolerability and safety and exploring the immunogenicity and therapeutic activity of AFFITOPE^®^ PD01A in PWP and GBA gene mutation
1 January 2017 to 1 July 2020	NCT02954978	Nilotinib	75	Impact of nilotinib on safety, tolerability, pharmacokinetics and biomarkers in PD (PD Nilotinib)
27 June 2017 to 14 September 2026	NCT03100149	RO7046015	316	A study to evaluate the efficacy of prasinezumab (RO7046015/PRX002) in participants with early PD (PASADENA)
16 October 2017 to 28 September 2019	NCT03205488	Nilotinib	76	Nilotinib in PD (NILO-PD)
17 October 2017 to 31 March 2021	NCT03272165	MEDI1341	50	Single ascending dose study of MEDI1341 in healthy volunteers
10 January 2018 to 29 April 2021	NCT03318523	BIIB054	357	Evaluating the efficacy, safety, pharmacokinetics, and pharmacodynamics of BIIB054 in participants with PD (SPARK)
25 July 2018 to 26 July 2021	NCT03611569	Lu AF82422	74	Lu AF82422 in healthy non-japanese and japanese subjects and in PWP
12 March 2019 to 23 April 2021	NCT03716570	BIIB054	24	A study to evaluate safety, tolerability, pharmacokinetics, and pharmacodynamics of BIIB054 in japanese participants with PD
4 August 2020 to 5 January 2022	NCT04449484	MEDI1341	25	Multiple ascending dose study of MEDI1341 in PWP
29 August 2019 to 1 March 2023	NCT04075318	UB-312	70	Study of UB-312 in healthy participants and PWP
29 August 2019 to 1 March 2023	NCT04075318	UB-312	70	Study of UB-312 in healthy participants and PWP
4 August 2020 to 5 January 2022	NCT04449484	MEDI1341	25	Multiple ascending dose study of MEDI1341 in PWP
22 December 2020 to 22 December 2022	NCT04685265	anle138b	70	A study to assess the safety, tolerability, pharmacokinetics and pharmacodynamics of Emrusolmin (anle138b and TEV-56286) in PD
5 May 2021 to 27 November 2026	NCT04777331	Prasinezumab	586	A study to evaluate the efficacy and safety of intravenous prasinezumab in participants with early PD (PADOVA)
1 May 2022 to 1 May 2023	NCT05355064	Trehalose	20	Efficacy of oral administration of trehalose in PWP
3 August 2022 to 4 December 2023	NCT05357989	Buntanetap/posiphen	523	A double-blind study to investigate efficacy and safety of buntanetap compared with placebo in participants with early PD
15 May 2023 to 1 January 2025	NCT05424276	IkT-148009	120	A randomized, double-blind, placebo-controlled trial of IkT-148009 in untreated PD
31 May 2023 to 1 May 2025	NCT05634876	UB-312	8	UB-312 in patients with synucleinopathies
24 July 2023 to 1 January 2028	NCT06015841	ACI-7104.056	150	A study to evaluate the effects of ACI-7104.056 vaccination in patients with early stages of PD (VacSYn)
18 March 2024 to 3 September 2024	NCT06258720	Lu AF82422	24	A trial investigating Lu AF82422 in healthy chinese and caucasian adults

Abbreviations: NA, not available/not applicable; PD, Parkinson’s disease; PWP, people with Parkinson’s disease.

## Data Availability

No new data created.

## Supplementary Materials

The following supporting information can be downloaded at: https://www.mdpi.com/article/10.3390/brainsci15121260/s1, Table S1: Pathological and Motor Phenotypic Characteristics Of αSyn Transgenic Mouse Models; Figure S1: Comprehensive schematic representation of sequential extraction of LB.

## Abbreviations

The following abbreviations are used in this manuscript:

AAV	Adeno-associated virus
AD	Alzheimer’s disease
ASO	Antisense Oligonucleotides
Aβ	Amyloid-β
BDNF	Brain-derived neurotrophic factor
CNS	Central nervous system
CSF	Cerebrospinal fluid
DLB	Dementia with Lewy bodies
DMV	Dorsal motor nuclei of the vagus
ECV	Extracellular vesicles
EM	Electron microscopy
GIT	Gastrointestinal tract
GWAS	Genome-wide association study
IL	Interleukin
LB	Lewy body
LRRK2	Leucine-rich repeat kinase 2
MSA	Multiple system atrophy
NAC	Non-amyloid-β component
NCT	National clinical trial
OM	Olfactory mucosa
PD	Parkinson’s disease
PDD	Parkinson’s disease dementia
PFF	Pre-formed fibril
PMCA	Protein misfolding cyclic amplification
PPMI	Parkinson’s progression markers initiative
PWP	People with Parkinson’s disease
RT-QuIC	Real-time quaking-induced conversion
SAA	Seed amplification assay
SMG	Submandibular gland
SN	Substantia nigra
SNP	Single nucleotide polymorphism
Tg	Transgenic
TH	Tyrosine hydroxylase
TLR	Toll-like receptor
TNF-α	Tumor necrosis factor α
αSyn	α-synuclein
