# Supplementary material for: Alpha-Synuclein Neurobiology in Parkinson’s Disease: A Comprehensive Review of Its Role, Mechanisms, and Therapeutic Perspectives
Source: Brain Sci. 2025 Nov 25;15(12):1260. doi: 10.3390/brainsci15121260 (PMC12731173; doi:10.3390/brainsci15121260)
Supplement: Supplementary file 1 [file brainsci-15-01260-s001.zip › brainsci-3975193-supplementary.pdf]

Supplementary Material

Tables

Table S1. Pathological and Motor Phenotypic Characteristics Of α-Synuclein Transgenic Mouse Models.

Figures

Figure S1. Comprehensive schematic representation of sequential extraction of LB.

Table S1. Pathological and Motor Phenotypic Characteristics Of αSyn Transgenic Mouse Models.

Mouse model	αSyn type	Expression patttern	Pathology	Motor phenotype	Age of onset	Reference
Thy1-αSyn (Line 61)	Human wild-type	Neurons (higher expression)	αSyn aggregation, synaptic dysfunction	Motor impairments (mild to moderate)	4–6 months	Roshanbin et al. (2021) [80]
Thy1-A53T	Human A53T mutant	Neurons	αSyn accumulation, axonal pathology, gliosis	Severe motor impairment, early death	2–6 months	Paumier et al. (2013) [81]
Thy1-A30P αSyn	Human A30P mutant	CNS neurons	αSyn aggregation, synaptic dysfunction, neurodegeneration	Progressive motor deficits (tremors, rigidity)	6–9 months	Ekmark-Lewén et al. (2018) [82]
PDGF-β (Line D)	Human wild-type	Neurons (widespread)	αSyn aggregates, synaptic dysfunction, neurodegeneration	Progressive motor deficits	6 months	Amschl et al. (2013) [83]
Tg(Camk2a-αSyn)	Human wild-type	Forebrain neurons (hippocampus, cortex)	αSyn inclusions, axonal degeneration	Cognitive impairment, mild motor dysfunction	8-12 months	Quadri et al. (2020) [84]
E46K αSyn (Line M47)	Human E46K mutant	Neurons	Early neurodegeneration, synaptic dysfunction	Early motor impairments, cognitive decline	4–6 months	Emmer et al. (2011) [85]
M83	Human A53T mutant	CNS neurons	Extensive αSyn inclusions, gliosis, neuronal loss	Paralysis, severe motor impairments	8–12 months	Bétemps et al. (2014) [86]

Abbreviations: αSyn, α-synuclein; BAC, bacterial artificial chromosome; CNS, Central Nervous System; Dpl, Doppel (a protein related to prion protein); PrP, Prion Protein; SNCA, Synuclein Alpha (the gene encoding alpha-synuclein); Thy1, Thy1 promoter (used to drive expression in neurons); Tg, Transgenic (refers to mice with inserted foreign genes); WT, Wild-type (refers to the normal, non-mutant form of a gene or protein).

Figure S1

Comprehensive schematic representation of sequential extraction of Lewy bodies

1. High Salt Extraction with HS/TX Buffer

1.A Preparation

Homogenize brain tissue in HS/TX buffer containing:
- Tris(hydroxymethyl)aminomethane (pH 7.4), NaCl (sodium chloride), NaF (sodium fluoride), EDTA (ethylenediaminetetraacetic acid), DTT (dithiothreitol), Triton X-100
- Protease and phosphatase inhibitors

1.B Centrifugation

- 180,000 xg for 30 minutes at 4°C
- Collect supernatant (HS-TX Total) and pellet (S1)

1.C Processing

- Resuspend pellet in HS/TX buffer (5 mL/g)
- Homogenize and keep on ice for 20 minutes
- Centrifuge at 180,000 xg for 30 minutes at 4°C
- Collect pellet (S2)

2. Myelin Floatation

- Add myelin floatation buffer to pellet (S2)
- Homogenize and keep on ice for 20 minutes
- Collect pellet (S3)

3. Sarkosyl Extraction

- Resuspend pellet in HS buffer with DTT
- Add 1% Sarkosyl (Sark)
- Homogenize (save aliquots as Sark-T)
- Incubate at room temperature (RT) for 1 hour
- Centrifuge at 180,000 xg for 30 minutes at 4°C
- Collect supernatant (Sark-Sup) and pellet

4. PBS Wash

- Resuspend pellet in sterile phosphate-buffered saline
- Sonicate and centrifuge at 1000 xg for 5 minutes
- Save supernatant at -80°C for storage

Figure S1. Comprehensive schematic representation of sequential extraction of LB.
